# Case Report: Humanized Selective CD19CAR-T Treatment Induces MRD-Negative Remission in a Pediatric B-ALL Patient With Primary Resistance to Murine-Based CD19CAR-T Therapy

**DOI:** 10.3389/fimmu.2020.581116

**Published:** 2020-12-23

**Authors:** Kai Wang, Yu Zhao, Xuan Wang, Bin Wang, Maoquan Qin, Guanghua Zhu, Huantong Wu, Zhongfeng Liu, Xueling Zheng, Huyong Zheng, Zhiguo Chen

**Affiliations:** ^1^ Beijing Key Laboratory of Pediatric Hematology Oncology, National Key Discipline of Pediatrics (Capital Medical University), Key Laboratory of Major Diseases in Children, Ministry of Education, Hematology Oncology Center, Beijing Children’s Hospital, Capital Medical University, National Center for Children’s Health, Beijing, China; ^2^ Cell Therapy Center, Beijing Institute of Geriatrics, Xuanwu Hospital Capital Medical University, National Clinical Research Center for Geriatric Diseases, and Key Laboratory of Neurodegenerative Diseases, Ministry of Education, Beijing, China; ^3^ Center of Neural Injury and Repair, Beijing Institute for Brain Disorders, Beijing, China

**Keywords:** CD19hsCAR-T, B-ALL, humanized scFv, selective domain, primary resistance, GVHD

## Abstract

**Background:**

CD19 chimeric antigen receptor T cell (CD19CAR-T) has shown great potential to treat acute B cell lymphoblastic leukemia (B-ALL) and B cell lymphoma, and most of anti-CD19 scFv are derived from murine antibody sequences. However, about 10–20% of B-ALL patients exhibit primary resistance to murine-based CD19CAR-T (CD19mCAR-T). Herein, we report that a humanized selective CD19CAR-T (CD19hsCAR-T) may offer a solution to this problem.

**Case Description:**

A 10-year old boy was diagnosed with high-risk B-ALL in Mar., 2013, and relapsed in Oct., 2018, after he underwent haplo-identical hematopoietic stem cell transplantation (HSCT) in 2017. The patient then received haplo-identical CD19mCAR-T infusions twice following induction chemotherapy with Vincristine, Dexamethasone and Asparaginase (VDL), but no response was observed. We further treated this patient with CD19hsCAR-T following chemotherapy with Vindesine, Idarubicin, Dexamethasone, and Pegylated Asparaginase (VDLD) plus bortezomib. The patient achieved minimal residual disease-negative (MRDneg) complete remission with incomplete hematopoietic recovery (CRi), and remained in CRi for more than 8 months with manageable side effect. The patient, unfortunately, died of unidentified pulmonary infection on Jan. 25 2020.

**Conclusion:**

CD19hsCAR-T may have the potential to induce remission in patients who are primarily refractory to CD19mCAR-T.

## Introduction

Chimeric antigen receptors (CARs) are genetically engineered receptors that couple an extracellular single-chain variable fragment (scFv) specific to a tumor associated antigen (TAA), to intracellular signaling domains leading to T cell activation; the transduced cytotoxic T lymphocytes can thus be re-directed and specifically recognize malignant cells expressing this TAA. CD19CAR-T therapy has proved to be an efficacious treatment for a majority of B-cell malignancies. Nevertheless, the scFv sequences of most published CD19CAR-T studies are designed on the basis of murine antibody sequences [FMC63- or SJ25C1-mAbs; (1–4)], and accumulating evidence has revealed that host immune responses can probably recognize the murine scFv and render subsequent infusions ineffective (5–8). Furthermore, around 10–20% patients do not respond to CD19mCAR-T (9), and the underlying reasons remain elusive. Whether immunogenicity of murine-derived scFv might have contributed to this needs to be investigated.

**Figure 1:**
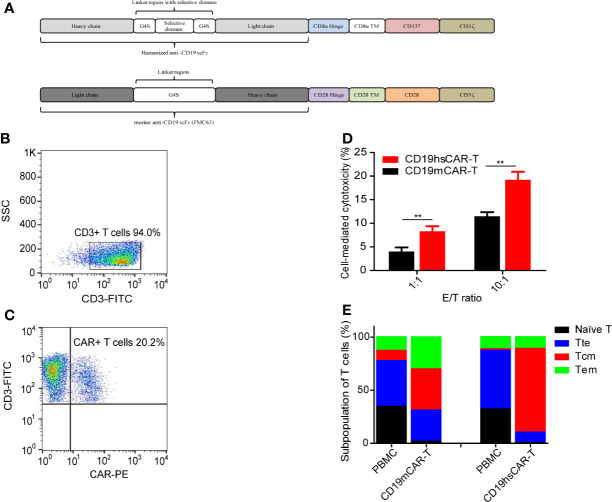
Features of CAR-T cells. **(A)** Structure of CD19hsCAR. CD19hsCAR contains a selective domain derived from human nuclear protein La/SBB that is inserted into the linker region between the heavy chain and light chain. The heavy chain and light chain are reversed in order with heavy chain placed in front. **(B, C)** Analysis of CD19hsCAR-T cells in the final product by flow cytometry. **(D)** Comparison of cellular cytotoxicity mediated by CD19mCAR-T and CD19hsCAR-T at different E/T ratios *in vitro*. Standard lactate dehydrogenase (LDH) release method was used to determine the cytotoxicity induced by CD19mCAR-T and CD19hsCAR-T, respectively. Target Raji cells (1×10^4) were incubated with the final product of CD19mCAR-T (culture of day 14) or CD19hsCAR-T (culture of day 10) at two effector/target (E/T) ratios (1:1 and 10:1) in 96-well microplates. After 12-hour cultivation at 37°C, the supernatant was harvested and cytotoxicity measured by using CytoTox 96 Non-Radioactive Cytotoxicity Assay Kit (Promega), following the manufacturer’s instructions. P value was determined by two-way ANOVA, **P<0.01. The data were presented as mean ± SD (n=3). **(E)** Subpopulations of T cells in final products of CD19hsCAR-T vs. CD19mCAR-T. The proportions of different T cell subpopulations in the starting PBMCs and final products of CD19mCAR-T and CD19hsCAR-T were analyzed. The proportion of the central memory T cells was enlarged in the final product. FP, final product; PBMC, peripheral blood mononuclear cells; Tte, terminal differentiated T cells; Tcm, central memory T cells; Tem, effector memory T cells.

We have previously reported a humanized CD19-specific CAR that incorporates a selective domain between the heavy and light chains, namely CD19hsCAR. CD19hsCAR possesses the following features: 1) a high affinity to CD19, 6-fold greater than that of murine-based CD19CAR (FMC63); 2) lower immunogenicity vs. murine-based counterpart; 3) a larger portion of central memory T cell subpopulation was obtained by stimulation with a monoclonal antibody specific to the selective domain (SmAb) during the production process (**Figure 1A**) (10). Accordingly, the clinical trial demonstrated that CD19hsCAR-T cells displayed a marked anti-tumor activity with mild side effect in heavily treated B-ALL patients who had relapsed from CD19mCAR-T-induced remission (10). Yet, whether CD19hsCAR-T is efficacious on patients who are primarily refractory to murine CD19CAR-T is still unknown.

Here, we report a case who failed to respond to CD19mCAR-T. In contrast, following CD19hsCAR-T treatment, the patient achieved MDRneg CRi and remained in CRi for around 8 months until he died of unidentified pulmonary infection in Jan., 2020.

## Case Description

**Figure 2:**
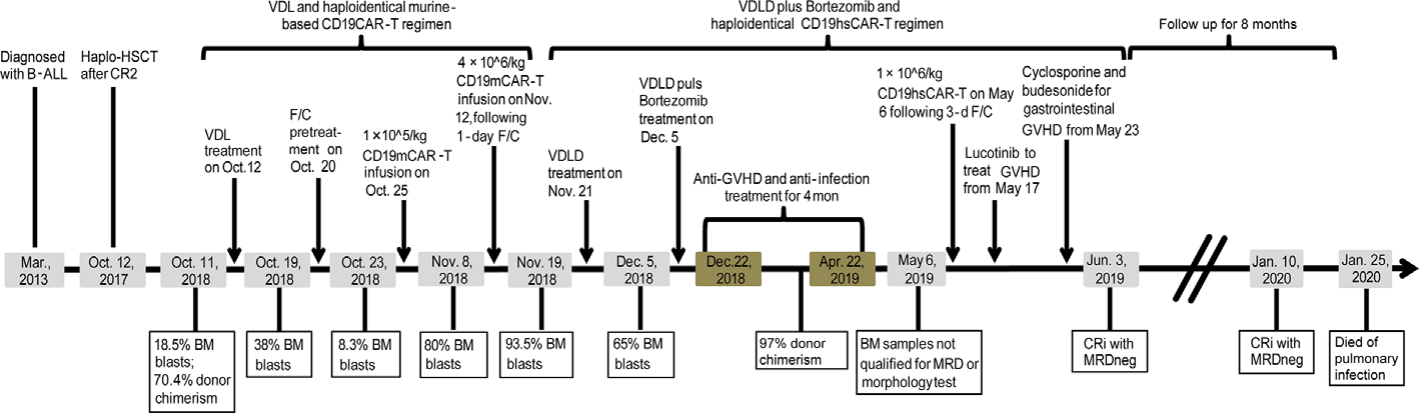
Time line of major treatments and outcomes for the patient.

**Figure 3:**
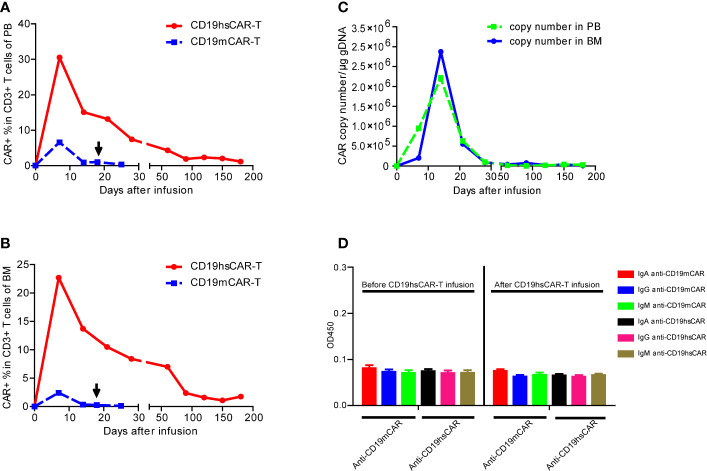
The expansion and persistence of CD19hsCAR-T in patient, and the anti-CAR immunoglobulins in the patient serum. **(A, B)** Expansion of CD19hsCAR-T (red dot solid line) and CD19mCAR-T (blue square dashed line) in PB and BM after infusion. The arrows indicate the time of the second infusion of CD19mCAR-T. **(C)** CD19hsCAR copy number in PB and BM was measured by using qPCR before and after infusion. PB, peripheral blood; BM, bone marrow. **(D)** Anti-CAR immunoglobulins, including IgA, IgG and IgM were examined before and after CD19 hsCAR-T infusion. The serum samples were collected at day 0 before and day 30 after CD19 hsCAR-T infusion. The data were presented as mean ± SE (n=3).

**Figure 4:**
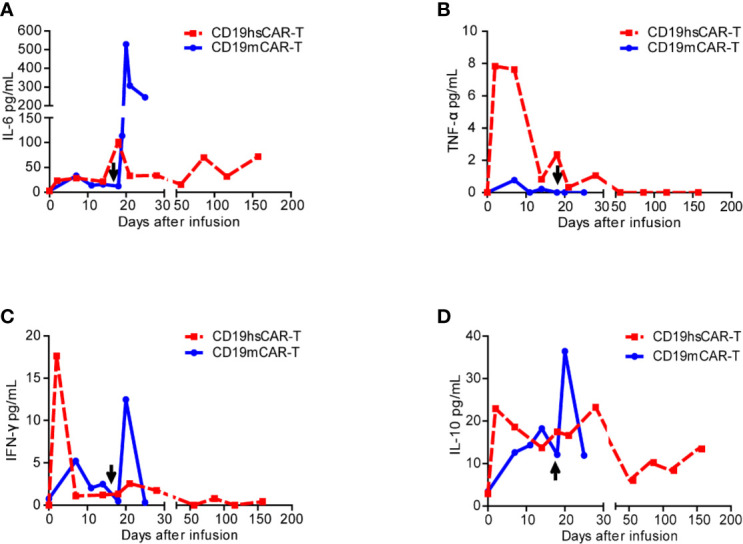
Cytokine levels in patient sera before and after infusions of CD19mCAR-T and CD19hsCAR-T. Various cytokines (**A**, IL-6; **B**, TNF-α; **C**, IFN-γ; **D**, IL-10) were detected by using ELISA before and after infusions of CD19hsCAR-T (red square dashed line) and CD19mCAR-T (blue dot solid line). The arrows indicate the time of the second infusion of CD19mCAR-T.

The study was registered at clinicaltrials.gov with a registration number NCT03902197. As shown in **Figure 2**, a male patient (born in May, 2009) was diagnosed with B-ALL in Mar., 2013, and achieved the first complete remission (CR1) with MRD testing positive after induction treatment in Apr., 2013; then the patient was subjected to the high-risk CCLG 2008 treatment protocol (11). In May, 2017, the patient relapsed one and a half years after the CCLG 2008 treatment had ceased. After three courses of chemotherapy, the patient achieved CR2 and then received haplo-identical hematopoietic stem cell transplantation (HSCT) from his mother as donor in Oct., 2017. The patient had remained in MRDneg CR for almost one year until the second relapse occurred in Oct., 2018, with the blasts being of the patient origin. Flow cytometry test revealed 18.5% of bone marrow (BM) blasts that expressed CD19, CD20, CD22, CD34, CD10, HLA-DR, CD56, and cyCD79a (**Table S1**; other major bone marrow test results were also included in **Table S1**). The donor chimerism rate was 70.4% (**Figure 2**). The patient was treated with VDL chemotherapy regimen (Vincristine 1.5 mg/m^2^ ×1, Dexamethasone 6 mg/m^2^/day for 7 consecutive days by oral administration, and Asparaginase 5,000 IU/m^2^ ×2) but no response was observed and the BM blasts continued to rise to 38%. Hence, the patient was given a CD19mCAR-T (**Figure 2**) treatment following a lymphodepleting preconditioning regimen (Fludarabine 25 mg/m^2^/d, for 3 days; Cyclophosphamide 250 mg/m^2^/d, days 1–3 for 3 days, F/C). Haplo-identical CD19mCAR-T cells (1×10^5^/kg) were infused on Oct. 25, 2018 (day 0) (The donor was the patient’s mother who was also his HSCT donor). Following treatment, the peak of percentage of CAR-T was detected as 6.63% and 2.43% in the peripheral blood (PB) and BM, on day 7 after infusion, respectively (**Figures 3A, B**). Meanwhile the percentage of peripheral B cells decreased from 11.3 to 0.8% (**Figure S1**). And the patient experienced grade 1 CRS as graded by the UPenn grading scale (12). However, the BM blasts increased to 80% on Nov. 8, 2018 (day 14 after the 1^st^ infusion), although the peripheral B cell level continued to go down to 0.1% (**Figure 2**, **Figure S1** and **Table S1**). At that time, the percentage of CAR-T decreased to 0.93% and 0.34% in PB and BM as detected by flow cytometry assay, respectively (**Figures 3A, B**). Considering that the first treatment dose might not be sufficient, the patient was given a second infusion of CD19mCAR-T at the dose of 4×10^6^/kg 4 days later on Nov. 12, 2018 (day 18 after the 1^st^ infusion) following a 1-day lymphodepleting F/C regimen. After the 2^nd^ infusion, no significant expansion of CAR-T was observed. The flow cytometry assay displayed that the peak values were 1.01% and 0.24% in PB and BM, respectively. The patient experienced grade 2 CRS with the peak concentrations of IL-6, IFN-γ and IL-10 being 539.14 pg/mL, 12.48 pg/mL and 36.48 pg/mL on day 7 after the 2^nd^ infusion, respectively (**Figures 4A–D**). Whereas, the BM blast load continued to rise to 93.5% as detected on Nov. 18, 2018 (day 25 after 1^st^ infusion/day 7 after the 2^nd^ infusion), and the peripheral CD19+ B cell percentage rose up from 0.1% to 4.5%, and then slightly decreased to 4.3% (**Figure S1**). The results suggested that the patient is primarily refractory to murine CD19CAR-T therapy. On Nov. 21, 2018, the patient started to be treated with modified VDLD regimen (Vindesine, 1.5mg/m^2^/week ×4; Idarubicin, 10mg/m^2^/week ×4; Dexamethasone, 6mg/m^2^/day for 28 days; Pegylated Asparaginase, 2,500 IU/m^2^/2 weeks ×2). The BM smear showed that there were still 65% immature lymphoblasts on Dec. 5, 2018, half way through the VDLD treatment. Subsequently, the patient received another sequential treatment - modified VDLD plus Bortezomib, with the plan to give a single dose of CD19hsCAR-T cells following this regimen. Bortezomib was administered (1.3 mg/m^2^/dose, 2 doses/week, × 4 doses) in combination with VDLD as described above. However, after the chemotherapy, the patient developed severe myelosuppression combined with sepsis caused by infection with enterococcus faecium and acinetobacter, and stage 4 hepatic graft-versus-host disease (GVHD) two weeks after treatment (bilirubin increased to 32.4 μmol/L on Dec. 26, 2018), and was unable to receive CD19hsCAR-T at that time. The GVHD was speculated to be related to donor T cells existing in the product of allogeneic CD19mCAR-T. For anti-infection treatment, the patient received infusion of 96 mL haplo-identical donor-derived BM, containing 0.13% CD34+ cells, on Jan. 2, 2019; and 120 mL haplo-identical donor-derived white blood cells on Jan. 3, 2019. The infused cell dosage, according to the body weight of patient (25kg), was 3.64 × 10^4^ CD34+ cells/kg and, 3.0 × 10^6^ CD3+ cells/kg, respectively. The hematopoietic parameters were recovered and infection was controlled about two weeks later. On Jan. 25, 2019, stage 4 gastrointestinal GVHD was observed, and basiliximab was administered (10 mg each time, repeated dosing at days 0, 4, 8 and 14), leading to an effective control of the symptom. Tumor burden was not tested because the patient’s BM samples did not qualify for smearing or flow cytometry assay during this period (from Dec. 22, 2018 to Apr. 30, 2019, **Figure 2**). After about 4 months of anti-GVHD therapy using methylprednisolone (2.5–5 mg/kg/day) plus cyclosporine (3–5 mg/kg/day), the patient was subjected to lymphodepleting F/C regimen (Fludarabine 25mg/m^2^/d, for 3 days; Cyclophosphamide 250mg/m^2^/d, for 3 days) started on May 1, 2019, in preparation for haplo-identical CD19 hsCAR-T treatment that was administered on May 6, 2019 (**Figure 2**). Given a high chimerism level of 97% as examined on Apr. 16, 2019, haplo-identical CD19hsCAR-T was generated by using the peripheral blood mononuclear cells (PBMCs) from the same donor of CD19mCAR-T (the donor was his mother, the same donor for his HSCT).

## Generation of CD19hsCAR-T Cells

CD19hsCAR-T cells were produced as previously described (10). Briefly, PBMCs from the donor were collected by leukapheresis. CD3-positive T lymphocytes were enriched and activated by using CD3/CD28 magnetic beads. Activated T cells were transduced with lentivirus expressing CD19hsCAR, and were stimulated by SmAb during the expansion stage *in vitro*. CD19hsCAR-T cells were harvested and cryopreserved after a 10-day culture. The final product was released for clinical administration after quality control test had passed. The structural feature of CD19hsCAR includes a humanized anti-CD19 scFv (referred to Patent US7635472B2) with a selective domain inserted between the heavy chain and light chain. A schematic representation of the CD19hsCAR was shown in **Figure 1A**. Flow cytometry analysis of the generated final product was shown in **Figures 1B, C**.

## Detection of CD19hsCAR-T Cells After Infusion

Flow cytometry was used to analyze the percentage of the CD19hsCAR-T cells in PB and BM samples with a biotin-labeled Protein-L (ACROBiosystems, USA) and PE Streptavidin antibody (Biolegend, USA). CD3-positive T cells were stained by using mouse anti-human CD3-FITC monoclonal antibody (Biolegend, USA). CD19hsCAR copy numbers were quantified by using qPCR as previously described (13).

## Cytokine Measurement

Serum levels of interleukin-2 (IL-2), -4 (IL-4), -6 (IL-6), and -10 (IL-10), tumor necrosis factor-α (TNF-α) and interferon-γ (IFN-γ) were analyzed by ELISA following the manufacturer’s instructions.

## Detection of Anti-Chimeric Antigen Receptor Immunoglobulins

CAR-specific immunoglobulins, including IgA, IgG and IgM were detected following the previous description (10). In brief, sera were collected from the patient before and after CD19hsCAR-T infusion. Recombinant extracellular domain of CD19mCAR and CD19hsCAR (1 mg/mL in PBS) were diluted to 4 μg/mL using 0.1 mmol/L PBS, and were coated to the bottom of 96-well ELISA plates. Test wells were blocked for 30 min at 37°C using 1% BSA. Samples (100 μL) were added into the wells and incubated for 1 h at 37°C. HRP-labeled goat-anti human antibodies specific for IgA, IgG, or IgM (Beijing Zhuang-Meng Biotechnology Co., Ltd) were added into the wells after 5 washes. TMB substrate was used for color development, and measurement was performed by using microplate reader at 450 nm. Positive results were set at an OD450 value >0.2.

## Modified Vindesine, Idarubicin, Dexamethasone, and Pegylated Asparaginase Plus Bortezomib Followed by CD19hsCAR-T Treatment Induces Durable MRDneg Complete Remission With Incomplete Hematopoietic Recovery

Prior to modified VDLD/Bortezomib and CD19hsCAR-T regimen, the BM examination of this patient showed 93.5% lymphoblasts in smear. After modified VDLD plus Bortezomib were administered as described above, unfortunately, stage 4 hepatic and gastrointestinal GVHD occurred in combination with severe infection. Anti-GVHD and anti-infection regimens were administered for about 4 months, until the patient was estimated to be able to tolerate CD19hsCAR-T therapy. Unfortunately, at this time (right before CD19hsCAR-T), the tumor burden test was not successfully performed in that the patient’s BM samples did not qualify for smearing or flow cytometry test. Infusion of CD19hsCAR-T was performed on May 6, 2019, at a dose of 1×10^6/kg, following the 3-day F/C pretreatment. After infusion, peripheral B cell percentage and tumor burden was monitored using flow cytometry and/or smearing at various intervals within one month without any additional anti-tumor treatment. According to the results, peripheral B cells decreased to undetectable level after 14 days from 1.5% on day 0, and the blast was not measurable at day 7 and day 14 after infusion (**Figure S1** and **Table S1**). The patient achieved MRDneg CRi by day 28 post-infusion. There was no evidence of blasts in BM either by BM smear or by flow cytometry at serial time points afterwards (8 months in CRi as to Jan. 10, 2020, **Table S1**). On Jan. 25, 2020, the patient died of severe pneumonia, an accident unrelated to CAR-T treatment.

## Expansion of CD19hsCAR-T and Durable Persistence In Vivo

After infusion, CD19hsCAR-T expanded and peaked at the level of 30.5% in the circulating T lymphocytes on day 7, followed by a contraction phase tapering off to 7.4% on day 28 (**Figure 3A**). Circulating B cells were also reduced to an undetectable level by day 28 and were maintained at this level thereafter as examined by flow cytometry (**Figure S1**). In BM, CD19hsCAR-T displayed similar expansion profile. The peak value (22.7% in the T lymphocytes) was reached on day 7 after infusion, and then decreased to 8.4% on day 28 (**Figure 3B**). CD19hsCAR copy number in PB and BM reached peak values on day 14 (**Figure 3C**). Persistence of CD19hsCAR-T was maintained after infusion, in agreement with the observed B cell aplasia. Six months after infusion, the proportions of CAR+ cells among T cells in PB and BM remained at 1.19% and 1.76%, respectively, and the copy number was 2.9×10^4/μg in genomic DNA in the circulating PB (**Figures 3A–C**).

## Toxicity of CD19hsCAR-T

After CD19hsCAR-T infusion, the patient experienced grade 1 CRS according to the UPenn grading scale (12). The temperature rose from 37.7 to 38.7°C on day 14 post-infusion, and then decreased and stayed below 38.0°C without any additional medical treatment. CRS-related cytokine IL-6 peaked up to 101.55 pg/mL on day 17 post-infusion, and IL-10 reached the peak value of 22.92 pg/mL on day 2. TNF-α and IFN-γ reached the peak value of 7.84 pg/mL and 17.63 pg/mL, respectively, on day 2 post-infusion. (**Figures 4A–D**). IL-2 and IL-4 were below detectable levels (data not shown).

On day 10 post-infusion, stage 3 acute cutaneous and hepatic GVHD was observed. Lucotinib was administered orally to alleviate GVHD. On day 20 post-infusion, gastrointestinal GVHD was appearing, and Cyclosporine and Budesonide were given for anti-GVHD treatment. The levels of total bilirubin (TBil) and direct bilirubin (DBil) increased from 5.99 μmol/L to the peak value of 32.06 μmol/L, and from 0.87 μmol/L to the peak value of 24.74 μmol/L, respectively, on day 21 post-infusion (**Figure S2**). After anti-GVHD management, those parameters were reduced and kept at a low level (close to baseline) from day 96 onward (**Figure S2** and **Table S2**). Accordingly, the patient exhibited chronic GVHD that mainly manifested as bloody stools and weight loss. Persistent thrombocytopenia with platelet count ranging from 16×10^9 to 68×10^9/L without platelet transfusion could be acknowledged as a manifestation of chronic GVHD in the setting of reconstitution of normal hematopoiesis (**Table S3**). The systematic immunosuppressive treatment was given to the patient by oral administration of Cyclosporine and Budesonide, in an attempt to keep the chronic GVHD under control.

## Examination of The Serum Level of CD19mCAR-T-Specific Antibody

To investigate the potential mechanism of the resistance to haplo-identical CD19mCAR-T, serum-derived anti-CAR immunoglobulins was tested by using ELISA before and after CD19hsCAR-T. Our previous study shows that anti-CD19mCAR IgA could be present and/or induced by repeated dosing of CD19mCAR-T (10). As shown in **Figure 3D**, however, the CD19mCAR-specific antibody failed to reach a cut-off value of positivity before and after CD19hsCAR-T infusion, suggesting that anti-CD19hsCAR immunoglobulins were not present in the patient’s sera. Furthermore, unfortunately, the serum samples before the 1^st^ and 2^nd^ CD19mCAR-T infusions were not available. The results indicated that resistance to CD19mCAR-T was probably not induced by humoral immunity.

## Discussion and Conclusion

In this case, we reported a refractory and relapsed B-ALL patient who showed primary resistance to haplo-identical murine-based CD19CAR-T, but achieved MRDneg CRi for more than 8 months after treatment with CD19hsCAR-T following modified VDLD plus Bortezomib. After CD19hsCAR-T vs. CD19mCAR-T treatment, the patient experienced milder side effect with grade 1 CRS and manageable GVHD. CD19hsCAR-T may offer a treatment option for those patients who are primary refractory to murine-based CD19CAR-T.

CAR-T is a promising therapy and two products targeting CD19 for treatment of leukemia and lymphoma have been approved by FDA in 2017 and 2018. Despite the remarkable efficacy (around 70–90% CR rate), primary resistance to CD19mCAR-T, relapse from CR, and non-response to repeated treatment(s) in relapsing patients, remain significant clinical problems (9, 14). After the first CD19mCAR-T treatment, 10–20% patients failed to achieve CR; for those who achieved CR, 30–60% patients relapsed within one year (15, 16). As to the current case, the patient did not respond to CD19mCAR-T. CD19mCAR-T exhibited poor proliferation and persistence compared with that of CD19hsCAR-T *in vivo*. Accordingly, the tumor burden rapidly rose to 80% on day14 after the first infusion, and continued to increase to 93.5% on day 7 after the second infusion (day 25 after the first infusion) (**Figure 2** and **Table S1**). The results are concordant with published reports from other groups and ours, with an emphasis on the insufficient efficacy of CD19mCAR-T on those patients who are primarily refractory or receive repeated infusion(s) after relapse (10, 14).

The potential mechanisms underlying the failure of response to repeated dosing of murine-based CAR-T are not well-known. One possibility may lie in the immune recognition of the murine scFv sequence in CD19 CAR. Our group reported that IgA that is specific to scFv of FMC63, could be detected in sera from B-ALL patients who had relapsed after receiving CD19mCAR-T; and the existence of mCAR-specific IgA may have rendered the second CD19mCAR-T treatment ineffective (10). Yet it remains unclear what might have accounted for the primary resistance in certain patients. Most patients that have not undergone HSCT employed autologous PB for generation of CAR-T. Abnormality of the lymphocytes from these patients may lead to suboptimal functions of CAR-T cells, such as poor expansion, a short period of persistence, and insufficiency in cytotoxicity (16, 17). However, in this case, both murine-based and humanized CAR-T were generated from the same healthy HSCT donor (patient’s mother), and hsCAR-T worked well, thus arguing against the possibility of source material abnormality. Furthermore, no immunoglobulin specific to CD19mCAR or CD19hsCAR was detected in the sera of the patient (**Figure 3D**), suggesting that, in this case, resistance to CD19mCAR was probably not caused by inhibitory component in the humoral immunity system. Yet the current results could not exclude the possibility that T cell-mediated immunity in host might have played a role in recognition of murine-based CD19 CAR. As an exogenous fusion protein, extracellular domain of CAR, especially murine-derived CARs, could possibly trigger T cell-based immunological rejection in which CD8+ T subpopulation particularly plays a pivotal role (5, 17, 18). Numerous clinical studies show that T cell response could be observed when murine-derived CAR-T was used to treat hematological malignances and solid tumors (19, 20). In this case, it is possible that T cell-mediated rejection to CD19mCAR-T could be a reason underlying treatment failure of CD19mCAR-T. Unfortunately, due to the limited amount of patient PB/BM samples, we could not perform such a test, and future studies with a large patient cohort are warranted to address this issue.

CD19hsCAR-T, in comparison with CD19mCAR-T, showed a different biological profile after infusion into the patient. The peak expansion of CD19hsCAR-T in PB and BM were around 5-fold (30.5 vs. 6.6%) and 10-fold (22.7 vs. 2.4%) greater than those of CD19mCAR-T on day 7 post-infusion, respectively (**Figures 3A, B**). On day 25 post-infusion, mCAR-T were almost undetectable in PB and BM, yet hsCAR-T was maintained at a level of 1.19% in PB and 1.76% in BM even 180 days after infusion (**Figures 3A, B**). The genomic DNA copy numbers of hsCAR were 2.9×10^4 and 9.9×10^4 in PB and BM, respectively, 180 days after infusion (**Figure 3C**). The results demonstrated a superior expansion and persistence of hsCAR-T vs. mCAR-T in patient.

Several possible reasons might have accounted for the superior clinical efficacy associated with hsCAR-T vs. mCAR-T. First, it may lie in the different biological structure/functions of the extracellular CARs. Not only including a humanized scFv, hsCAR also exhibits an affinity to CD19 6-fold greater than that of mCAR (FMC63) (10); with a higher affinity, the same dosage of hsCAR-T may exert a better efficacy than does mCAR-T, as indicated by the cytotoxicity assay results *in vitro* (**Figure 1D**). The fact that haplo-identical CD19mCAR-T cells showed normal cytotoxicity (**Figure 1D**) also suggested that the failure of CD19mCAR-T to treat this patient was not resulted from dysfunction of the mCAR-T cells *per se*. Second, due to the inclusion of selective domain and stimulation with SmAb during the production stage, hsCAR-T final products normally consist of a much higher proportion of central memory T cell subpopulation (**Figure 1E**) (10). Studies have shown that memory T cells are closely related to the anti-tumor potency, expansion and/or persistence of T cells (21–23). Difference in co-stimulatory domains could be a third reason. As shown in **Figure 1A**, 4-1BB-derived intracellular domain was used in CD19hsCAR versus the counterpart derived from CD28 was used in CD19mCAR. It has been demonstrated that 4-1BB domain signaling could prolong the persistence by regulating multiple cellular signaling pathways, such as those involved in increasing oxidative metabolism, upregulating the expression of the antiapoptotic genes, and enhancing the differentiation into central memory, compared with that of CD28 (24–26). Furthermore, CAR with CD28 co-stimulatory domain is often associated with a more rapid exhaustion after tonic activation of CAR *in vivo* compared with 4-1BB (27). In short, the structure of hsCAR offers some advantages that might have contributed to the superior clinical efficacy.

Another merit associated with CD19hsCAR-T as observed in this case was reduced side effect. Under similar clinical regimens, CD19hsCAR-T induced more manageable side effect, including transient fever, low-grade CRS and controllable GVHD, compared to those associated with previous CD19mCAR-T treatments. In this case, CD19hsCAR-T vs. CD19mCAR-T elicited lower peak levels of life-threatening CRS-related cytokines IL-6 (101.55 pg/mL vs. 529.14 pg/mL) and IL-10 (22.92 pg/mL vs 36.4 pg/mL); yet the peak levels of proinflammatory cytokines with anti-tumor activity were higher, such as TNF-α (7.84 pg/mL vs. 0.77 pg/mL) and IFN-γ (17.63 pg/mL vs. 12.48 pg/mL) (**Figure 4**). This clinical benefit may also be at least partly attributed to the construction of CD19hsCAR-T.

Infusion-related GVHD is another major side effect associated with administration of allogeneic/haplo-identical CAR-T for patients who relapsed after receiving HSCT. In this case, infusion-induced GVHD combined with infection was observed following both CD19mCAR-T and CD19hsCAR-T treatments. However, the severity and duration of GVHD were milder and shorter as associated with hsCAR-T vs. mCAR-T. Final product of CAR-T cells generated from allogeneic donors includes CAR-positive and CAR-negative subpopulations, and the CAR-transduction efficiency normally ranges from 10 to 60%. Ghosh et al. reported that cumulative activation of CAR and allo-reactive T cell receptor (TCR) leads to T cell exhaustion, leaving alive only those CAR-T cells with TCRs not reactive to host (28). Stimulation with CAR or allo-reactive TCR alone may not be sufficient to cause exhaustion, suggesting that the CAR-negative T cells may be a contributor to GVHD. By stimulation with SmAb, we can selectively expand the CAR-transduced T cells, resulting in a higher proportion of CAR-positive T cells in final product (10). A slightly smaller proportion of non-transduced T cells in allogeneic hsCAR-T vs. mCAR-T product might be one (but obviously not all) contributing factor to the milder GVHD side effect (**Table S4**). If this is true, using magnetic beads-conjugated SmAb to further purify the CAR-positive T cells in final product, may be an approach to reduce GVHD to a lower level, and this will be tested in our future studies. Other possible reasons for the mild side effect may include the higher affinity to CD19 and/or the relative lower dose (1×10^6/kg hsCAR-T vs. 4×10^6/kg mCAR-T in the second infusion). Nevertheless, in our previous study, 3×10^6/kg dosage of allogeneic hsCAR-T was used in some patients and the related side effects were also mild (10).

In a small proportion of B-ALL patients who had received HSCT and then relapsed, donor lymphocyte infusion (DLI) may induce a CR (29). However, this was probably not the reason accountable for the CR as observed after hsCAR-T treatment. The mCAR-T cells given prior to hsCAR-T included a majority of CAR-negative lymphocytes (**Table S4**); yet the donor lymphocytes in mCAR-T cells did not lead to CR. Also, after mCAR-T treatment, the patient received infusion of haplo-identical white blood cells and BM for control of infection; but still, such treatment did not induce a CR. These results suggest that DLI may not be a reason for CR in this case.

Furthermore, considering the rapid increase of tumor burden after failure of CD19mCAR-T treatment, bortezomib combined with induction/reinduction chemotherapy (VDLD) was used to control the disease progression, prior to CD19hsCAR-T infusion. As a first-generation 26S proteasome inhibitor, bortezomib has displayed significant anti-tumor effects in treatment of advanced B-ALL in clinical trials, but the combined chemotherapy protocols and the target patient population reported in those trials were quite different than our study (30). August et al. reported a clinical study in which patients with advanced B-ALL (relapse 2^nd^ and above) were enrolled and received the treatment of bortezomib plus VLXMtz (Mitoxantrone, Vincristine, PEG-aspargase, Dexamethasone, and intrathecal methotrexate). Similar to the current case, 4 B-ALL patients received both HSCT and CAR-T treatment previously in that clinical trial, but it was unclear whether HSCT was bridged to CAR-T therapy. After treatment, 3 of those 4 patients showed response (2/3 was CR, 1/3 was PR), and the range of duration was from 7 to17 months (31). Notably, all of the 3 patients also received further consecutive therapies including HSCT and CAR-T for disease control. Therefore, the efficacy and durability of bortezomib plus standard induction/reinduction chemotherapy alone still need to be further validated. As to the current case, at least it suggests that CD19hsCAR-T following bortezomib/VDLD might be employed as a treatment regimen for those patients refractory to CD19mCAR-T. Considering the marked expansion and persistence of hsCAR-T, and the maintenance of undetectable B cell level, we thought the 8-month CRi status could be, more likely, attributed to CD19hsCAR-T therapy.

In summary, haplo-identical CD19hsCAR-T was administered to treat a highly progressive pediatric B-ALL patient who showed primary resistance to murine-based CD19CAR-T. Following hsCAR-T treatment, MRDneg CRi was achieved and maintained for more than 8 months with manageable GVHD before the patient died of unrelated accident. The study suggests that CD19hsCAR-T might offer an option for the heavily treated B-ALL patients, particularly those who are refractory to or have relapsed from murine-based CD19CAR-T therapy.

## Data Availability Statement

The original contributions presented in the study are included in the article/**Supplementary Material**; further inquiries can be directed to the corresponding author/s.

## Ethics Statement

The studies involving human participants were reviewed and approved by Institutional Review Board of Beijing Children’s Hospital, Capital Medical University. Written informed consent to participate in this study was provided by the participants’ legal guardian/next of kin.

## Author Contributions

KW, BW, MQ, GZ, and HZ managed the clinical protocol and treated the patient. KW and XZ collected the clinical data. YZ, XW, HW, ZL, and ZC produced the CD19hsCAR-T cells and performed related analysis. HZ and ZC designed and guided the study. YZ, XW, KW, ZC, and HZ reviewed the data and wrote the paper. All authors contributed to the article and approved the submitted version.

## Funding

This work was supported by the Beijing Municipal Administration of Hospitals DengFeng Program (Grant # DFL20151101), Capital Health and Development of Special Grant (Grant # 2016-1-2091), and National Science and Technology Key Projects (Grant # 2017ZX09304029004) to HZ, and grants from Stem Cell and Translation National Key Project (2016YFA0101403), National Natural Science Foundation of China (81973351, 81661130160, 81422014, 81561138004), Beijing Municipal Natural Science Foundation (5142005), Beijing Talents Foundation (2017000021223TD03), Support Project of High-level Teachers in Beijing Municipal Universities in the Period of 13th Five-Year Plan (CIT & TCD20180333), Beijing Medical System High Level Talent Award (2015-3-063), Beijing Municipal Health Commission Fund (PXM 2018_026283_000002), Beijing One Hundred, Thousand, and Ten Thousand Talents Fund (2018A03), and the Royal Society-Newton Advanced Fellowship (NA150482). This Study was also supported by the Beijing Municipal Administration of Hospitals DengFeng Program (No. DFL20151101), Capital Health and Development of Special Grant (No. 2016-1-2091), and National Science and Technology Key Projects (No. 2017ZX09304029004) to ZC.

## Conflict of Interest

The authors declare that the research was conducted in the absence of any commercial or financial relationships that could be construed as a potential conflict of interest.

## Abbreviations

B-ALL, acute B cell lymphoblastic leukemia; BM, Bone marrow; CARs, Chimeric antigen receptors; CD19CAR-T, CD19 chimeric antigen receptor T cell; CD19hsCAR-T, Humanized selective CD19CAR-T; CD19mCAR-T, Murine-based CD19CAR-T; CR, Complete remission; CRi, CR with incomplete hematopoietic recovery; CRS, Cytokine release syndrome; DLI, donor lymphocyte infusion; F/C, Chemotherapy with fludarabine and cyclAophosphamide; GVHD, Graft-versus-host disease; Haplo-HSCT, Haplo-identical hematopoietic stem cell transplantation; HLA-DR, human leukocyte antigen DR; IFN-γ, Interferon-γ; IL-2, Interleukin-2; MRDneg, Minimal residual disease negative; PB, Peripheral blood; PBMCs, Peripheral blood mononuclear cells; scFv, Single-chain variable fragment; SmAb, Monoclonal antibody specific to selective domain; TAA, Tumor associated antigen; TCR, T cell receptor; TNF-α, Tumor necrosis factor-α; VDL, Chemotherapy with Vincristine, Dexamethasone and Asparaginase; VDLD, Chemotherapy with Vindesine, Idarubicin, Dexamethasone, and Pegylated Asparaginase.
