# Supplementary material for: Case Report: Humanized Selective CD19CAR-T Treatment Induces MRD-Negative Remission in a Pediatric B-ALL Patient With Primary Resistance to Murine-Based CD19CAR-T Therapy
Source: Front Immunol. 2020 Dec 23;11:581116. doi: 10.3389/fimmu.2020.581116 (PMC7786099; doi:10.3389/fimmu.2020.581116)
Supplement: Supplementary file 1 [file DataSheet_1.pdf]

Supplementary figure legend

Supplementary figure 1. Circulating B cell percentages in patient. Peripheral B cells were analyzed at different time points before and after CD19mCAR-T (blue square solid line) and CD19hsCAR-T (red dot dashed line) infusion. The arrow indicates the second infusion of CD19mCAR-T.

Supplementary figure 2. Changes of bilirubin levels following treatment with CD19hsCAR-T. Total bilirubin (TBil, red dot solid line) and direct bilirubin (DBil, blue square dashed line) levels in patient's sera were measured at a series of indicated time points.

Figure S1

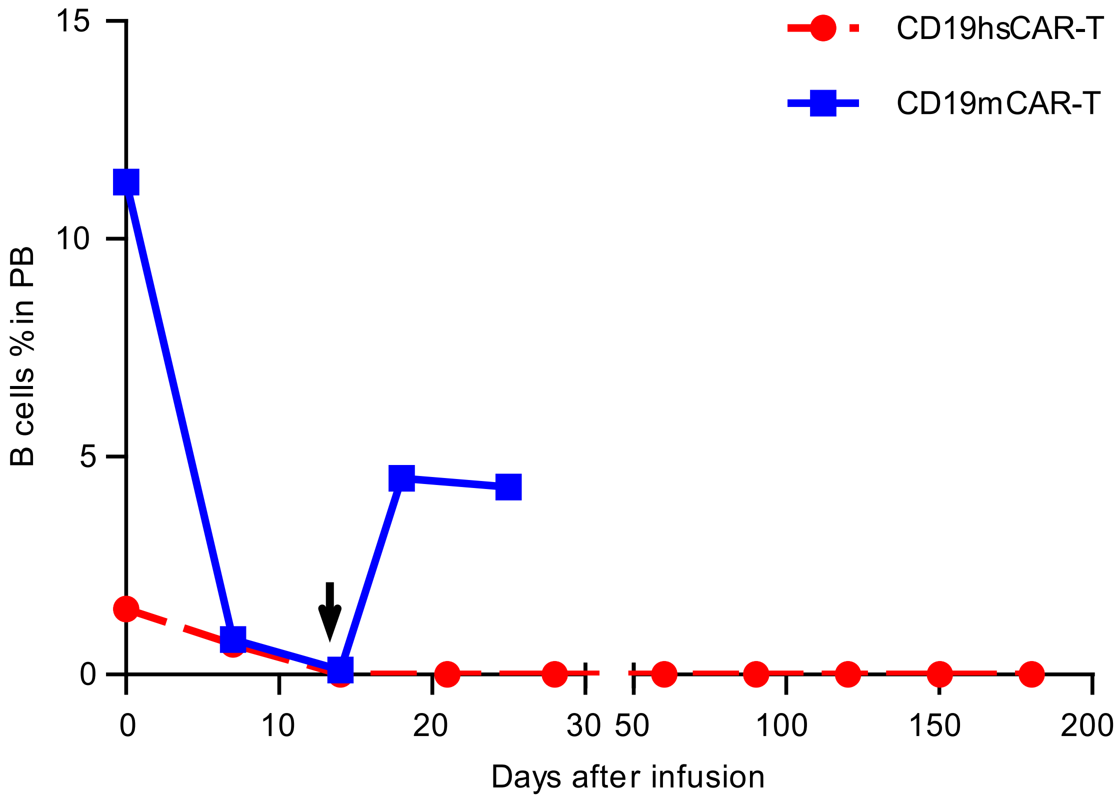

CD19hsCAR-T
CD19mCAR-T
B cells % in PB
15
10
5
0
0
10
20
30
50
100
150
200
Days after infusion

Figure S2

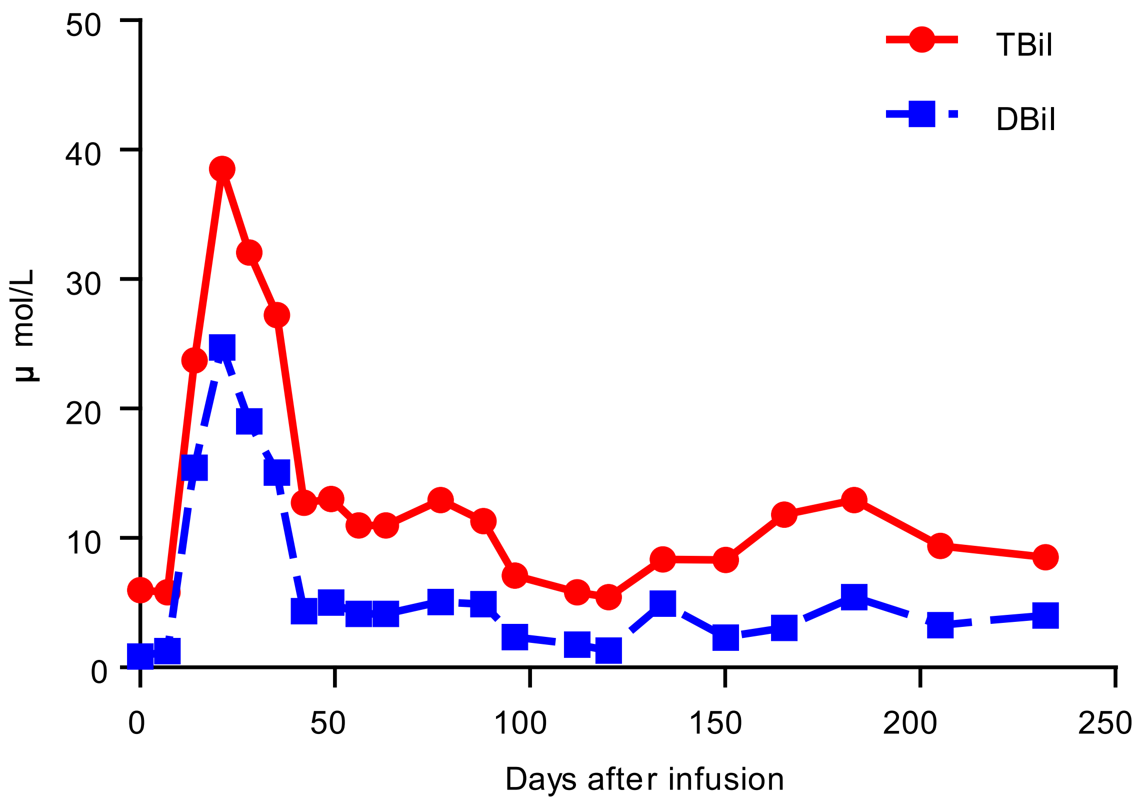

50
40
30
20
10
0
μ mol/L
0
50
100
150
200
250
Days after infusion
TBil
DBil

Table S1. Clinical test results of bone marrow samples.

Date	Major results of bone marrow samples	Note
2018.10.11	1. Hyperplasia of bone marrow was actively ongoing.<br>2. Lymphoblast and prolymphocyte total count was 16.5% (18.5% by flow cytometry).<br>3. Granulopoiesis was partially arrested.<br>4. Erythropoiesis was generally normal.<br>5. Megakaryocytes and platelets were occasionally observed.	The second relapse was initially detected one year after HSCT
2018.10.19	1. Lymphoblasts and prolymphocytes total count was 38% (flow cytometry not performed).<br>2. Granulopoiesis was abnormal.<br>3. Megakaryocytes were not observed.<br>4. Platelets were occasionally observed.	Day 7 after VDL chemotherapy
2018.10.23	1. Lymphoblasts and prolymphocytes total count was 8.3% (flow cytometry was not performed).<br>2. No nucleated granulocytes were observed.<br>3. The ratio of polychromatic normoblasts to orthochromatic normoblasts was low.<br>4. Megakaryocytes and platelets were not observed.	Day -1 relative to the day of the first CD19mCAR-T infusion
2018.11.08	1. Hyperplasia of bone marrow was actively ongoing.<br>2. Lymphoblasts and prolymphocytes total count was 80% (flow cytometry not performed).<br>3. Granulopoiesis and erythropoiesis were reduced.<br>4. Megakaryocytes were not observed.<br>5. Platelets were occasionally observed.	Day 14 after the first CD19mCAR-T infusion
2018.11.19	1. Hyperplasia of bone marrow was at a significant level.<br>2. Lymphoblasts and prolymphocytes total count was 93.5% (flow cytometry not performed).<br>3. Granulopoiesis and erythropoiesis were arrested.<br>4. Megakaryocytes and platelets were occasionally observed.	Day 8 after the second CD19mCAR-T infusion
2018.12.05	1. Hyperplasia of bone marrow was reduced.<br>2. Lymphoblasts and prolymphocytes total count was 65% (flow cytometry not performed).	Start treatment with VDLD plus bortezomib

	3. Granulopoiesis and erythropoiesis were arrested.<br>4. Megakaryocytes and platelets were occasionally observed.	
2019.05.06	Bone marrow sample did not qualify for MRD or morphology test.	Day 0 of CD19hsCAR-T infusion
2019.05.13	1. The observed granulocytes were mainly at the mature stage, and some neutrophils showed stained particles in cytoplasm.<br>2. Lymphoblasts and prolymphocytes were not observed (MDR-negative by flow cytometry).<br>3. Megakaryocytes were not observed.<br>4. Platelets were occasionally observed.	Day 7 after CD19hsCAR-T infusion
2019.05.20	1. The observed granulocytes were mainly at the mature stage, and some neutrophils showed stained particles in cytoplasm.<br>2. Lymphoblasts and prolymphocytes were not observed (MDR-negative by flow cytometry).<br>3. Nucleated erythrocytes were occasionally observed.<br>4. Megakaryocytes were not observed.<br>5. Platelets were occasionally observed.	Day 14 after CD19hsCAR-T infusion
2019.06.03	1. The observed granulocytes were mainly at the mature stage, and some neutrophils showed stained particles in cytoplasm.<br>2. Lymphoblasts and prolymphocytes were not observed (MDR-negative by flow cytometry).<br>3. The ratio of polychromatic normoblasts to orthochromatic normoblasts was low.<br>4. Megakaryocytes were not observed.<br>5. Platelets were occasionally observed.	About 1 month after CD19hsCAR-T infusion
2019.07.03	1. Hematopoiesis of bone marrow was active.<br>2. Lymphoblasts and prolymphocytes were not observed (MDR-negative by flow cytometry).<br>3. Granulopoiesis was generally normal.<br>4. Erythropoiesis was generally normal.<br>5. Megakaryocytes and platelets were occasionally observed.	About 2 months after CD19hsCAR-T infusion

2019.08.02	1. Hematopoiesis of bone marrow was active.<br>2. Lymphoblasts and prolymphocytes were not observed (MDR-negative by flow cytometry).<br>3. Granulopoiesis was normal.<br>4. Erythropoiesis was normal.<br>5. Megakaryocytes were not observed.<br>6. Platelets were occasionally observed.<br>7. Reticulocytes were observed.	About 3 months after CD19hsCAR-T infusion
2019.09.02	1. Hematopoiesis of bone marrow was active.<br>2. Lymphoblasts and prolymphocytes were not observed (MDR-negative by flow cytometry).<br>3. Granulopoiesis was normal.<br>4. Erythropoiesis was normal.<br>5. Megakaryocytes and platelets were occasionally observed.	About 4 months after CD19hsCAR-T infusion
2019.10.11	1. Lymphoblasts and prolymphocytes were not observed (MDR-negative by flow cytometry).<br>2. The observed granulocytes were mainly at the mature stage, and some neutrophils showed stained particles in cytoplasm.<br>3. Erythropoiesis was normal.<br>4. Megakaryocytes and platelets were occasionally observed.	About 5 months after CD19hsCAR-T infusion
2019.11.20	1. Hematopoiesis of bone marrow was active.<br>2. Lymphoblasts and prolymphocytes were not observed (MDR-negative by flow cytometry).<br>3. Granulopoiesis was normal.<br>4. Erythropoiesis was normal.<br>5. Megakaryocytes and platelets were occasionally observed.	About 6 months after CD19hsCAR-T infusion
2019.12.16	1. Hematopoiesis of bone marrow was active.<br>2. Lymphoblasts and prolymphocytes were not observed (MDR-negative by flow cytometry).<br>3. Granulopoiesis was normal.<br>4. Erythropoiesis was normal.	About 7 months after CD19hsCAR-T infusion

	5. Megakaryocytes were not observed.<br>6. Platelets showed scattered distribution.	
2020.01.10	1. Hematopoiesis of bone marrow was active.<br>2. Lymphoblasts and prolymphocytes were not observed (MDR-negative by flow cytometry).<br>3. Granulopoiesis was normal.<br>4. Erythropoiesis was normal.<br>5. Megakaryocytes were not observed.<br>6. Platelets were occasionally observed.	About 8 months after CD19hsCAR-T infusion

Table S2. Bilirubin test results.

Days after infusion	Total bilirubin µmol/L	Direct bilirubin µmol/L
0	5.990	0.87
7	5.810	1.25
14	23.730	15.42
21	38.510	24.74
28	32.060	19.00
35	27.220	15.08
42	12.730	4.36
49	13.020	5.01
56	10.980	4.15
63	10.980	4.15
77	12.920	5.05
88	11.300	4.88
96	7.120	2.37
112	5.830	1.77
120	5.430	1.32
134	8.350	4.94
150	8.320	2.31
165	11.810	3.06
183	12.950	5.44
205	9.412	3.29
232	8.540	4.03

Table S3. Platelet and white blood cell counts.

Days after infusion	Platelet count (10^9/L)	White blood cells (10^9/L)
0	31	4.22
7	21	5.34
14	68	7.24
21	23	5.49
28	16	6.76
58	26	5.48
88	19	4.54
118	26	5.96
178	17	3.13
210	35	3.85
236	43	4.37

Table S4. Subpopulation analysis of the final products.

		Final products	
		**CD19mCAR-T**	**CD19hsCAR-T**
**Starting PBMCs**	**CD3+ in PBMCs**	8.31%	6.79%
	**CD19+ in PBMCs**	1.94%	2.41%
	**Naïve T cells in CD3+ T cells**	34.56%	32.15%
	**Terminal differentiated T cells in CD3+ T cells**	43.00%	55.06%
	**Central memory T cells in CD3+ T cells**	9.54%	1.34%
	**Effector memory T cells in CD3+ T cells**	12.90%	11.45%
**Final Product (FP)**	**CD3+ in FP**	95.2%	94.0%
	**CD19+ in FP**	OOL	OOL
	**CAR+ T cells in FP**	15.7%	20.2%
	**Naïve T cells in CAR+ T cells**	1.80%	0.45%
	**Terminal differentiated T cells in CAR+ T cells**	29.40%	9.89%
	**Central memory T cells in CAR+ T cells**	38.70%	78.66%
	**Effector memory T cells in CAR+ T cells**	30.10%	11.00%

Note: FP, final product; OOL, out of limit.
